# Rotavirus and illness severity in children presenting with acute gastroenteritis at the primary care out-of-hours service

**DOI:** 10.1080/13814788.2021.2011205

**Published:** 2021-12-13

**Authors:** Pien Wolters, G. A Holtman, A. A. H Weghorst, M. Knoester, M. Y. Berger

**Affiliations:** a Department of General Practice and Elderly Care Medicine, University of Groningen, University Medical Centre Groningen, Groningen, The Netherlands; b Department of Medical Microbiology and Infection Prevention, University of Groningen, University Medical Centre Groningen, Groningen, The Netherlands

**Keywords:** After-hours care, faeces/virology, gastroenteritis/virology, paediatrics, primary care

## Abstract

**Background:**

Rotavirus is a common cause of acute gastroenteritis in young children in the Netherlands, where rotavirus vaccination has not yet been implemented.

**Objectives:**

To evaluate a difference in illness severity course depending on the presence of rotavirus infection and assess the prevalence of viruses and the referral rate in children with acute gastroenteritis.

**Methods:**

A prospective cohort of children aged 6 months to 6 years presenting with acute gastroenteritis to a primary care out-of-hours service from October 2016 to March 2018. Faeces were sampled and sent to a laboratory where viral pathogens were identified and quantified by real-time polymerase chain reaction. Severe course of acute gastroenteritis was defined as a Modified Vesikari Score of ≥11. In addition, we assessed referral rates. Chi-square tests were used to evaluate differences between groups.

**Results:**

We included 75 children (34 boys) with a median age of 1.5 years (interquartile range, 0.9–2.0 years). The prevalence of rotavirus was 65.3% (95% confidence interval, 53.5–76.0) with a median cycle threshold of 16.0. Severe course of acute gastroenteritis was present in 31 of 71 children (4 were lost to follow-up). Those with rotavirus (20/47) did not have a severe course more often than those without (11/24): odds ratio, 0.88 (95% confidence interval, 0.33–2.36). Referral rates were comparable for rotavirus (15.2%) and non-rotavirus (14.3%).

**Conclusion:**

In out-of-hours primary care, rotavirus is common but not associated with increased severity and higher referral rates in children with acute gastroenteritis.

KEY MESSAGES

- In children with acute gastroenteritis in out-of-hours primary care, rotavirus is the most often detected pathogen
- In out-of-hours primary care we could not show a significant association between rotavirus and severe course
- The fact that rotavirus is not associated with severe course corresponds with referral rates being comparable between groups.

## Introduction

Acute gastroenteritis is a common infectious disease, especially in children [1]. Typically, it has an uncomplicated and self-limiting course, allowing it to be managed with simple advice and instructions from a general practitioner (GP) [2]. However, acute gastroenteritis is also a major cause of hospital admission for children worldwide if it follows a more severe course that results in dehydration and requires hospitalisation [2]. These admissions not only burden the child and his or her parents but also result in considerable costs that are borne by the health care system and each household [3–5].

It is difficult to estimate the extent of dehydration based on clinical signs and symptoms, making it difficult for a GP to predict the disease course and to provide optimal medical care. Knowing the causative pathogen can provide some insight into the general course and could help GPs rationalise management. Specifically, in young children, rotavirus and norovirus are frequent causes of acute gastroenteritis [6,7]. Compared with other infections, rotavirus more often leads to a severe course in children hospitalised for acute gastroenteritis, typically with symptoms of heavy vomiting, fever, severe watery diarrhoea, and a higher risk of severe dehydration and even death [3,7–10]. By contrast, acute gastroenteritis due to norovirus is usually self-limiting and presents with milder symptoms [9,11–13].

Although much is known about the causative pathogens in severe acute gastroenteritis among children in both primary and secondary care, little is known in primary care out-of-hours (OOH) settings. This knowledge is important because it could help when differentiating children at high and low risk for dehydration, and as such, could help to prevent some referrals to secondary care [14]. This is particularly relevant in a setting where a patient’s usual continuity of care may have been lost, such as in the OOH setting. Improved knowledge about pathogens in this setting could also have implications for introducting a point-of-care test or a vaccine for rotavirus.

This study aimed to investigate if a difference in illness severity existed depending on the presence or absence of rotavirus infection. We also evaluated the prevalence of rotavirus and other viruses and the referral rate in young children with acute gastroenteritis presenting OOH in primary care.

## Methods

### Study design

This prospective cohort study was performed from October 2016 to March 2018 at three primary care OOH services in the north of the Netherlands. All parents of the included children gave written informed consent, and the Medical Ethics Review Committee of the University Medical Centre Groningen approved the study (register number 201600704).

### Setting

The OOH service in primary care. Given that the prevalence of acute gastroenteritis in children visiting the GP-practice in daytime and in OOH service is similar [15], the latter setting made it easier to include patients of more different regions with a limited number of inclusion locations and research assistants.

### Study population

We consecutively included children if they met the inclusion criteria, which were an age of 6 months to 6 years and a GP-confirmed diagnosis of acute gastroenteritis. Exclusion criteria were lack of parental consent or participation in a parallel randomised controlled trial which included children with acute gastroenteritis and severe vomiting [16]. The age range was chosen to coincide with the peak incidence of acute gastroenteritis in children and because younger children are at higher risk of dehydration and a complicated course [11,17].

### Data collection

Prior to consultation with the GP, a research assistant (RA) informed the parents and the child (if appropriate) about the study. The RA then performed baseline measurements and collected patient data in a case report form, including the child’s demographics, medical history, vital parameters (e.g. temperature, heart rate, capillary refill time, skin turgor, and weight), and symptoms (e.g. frequency and duration of diarrhoea and vomiting). GP care was then free to continue as usual.

Parents were asked to collect a stool sample at home, place the sample in a special envelope, and return it to the University Medical Centre Groningen by mail. We instructed them to take the sample as soon as possible and store it in the refrigerator until returned. There were no limits to collecting and sending the sample. All parents were also asked to keep a diary in which we enquired about the course of the acute gastroenteritis after visiting the GP (e.g. the frequency of diarrhoea and vomiting, if referred to a paediatric department). The diary needed to be completed hourly for the first 4 h after visiting the GP but thereafter, only needed to be completed daily for the 7-day follow-up. The RA called parents to remind them to complete the diary 3 days after inclusion.

We asked parents to return the diary by mail to the University Medical Centre Groningen. If no diary had been received a month after inclusion, and after three telephone reminders (at 1, 2 and 4 weeks), the child was considered lost to follow-up. We excluded children from whom we received no stool sample.

### Pathogen testing

Multiplex real-time polymerase chain reaction was used to test stool samples for viral, bacterial, and parasitic pathogens [18], including adenovirus, astrovirus, enterovirus, norovirus, rotavirus, sapovirus, *Campylobacter spp.*, *Salmonella spp.*, *Shigella spp.*, *Cryptosporidium*, and *Giardia lamblia*. The cycle threshold (Ct) value was recorded for detected pathogens. This was used to quantify the viral, bacterial, or parasitic load, with lower values indicating a higher load. The pathogen was considered to be present in high amounts if the Ct value was <20 (strongly positive); in medium amounts if between 20 and 30 (moderately positive); and in low amounts if >30 (weakly positive) with a cut off value of 40 for all targets.

### Outcomes

Our primary outcome focussed on a severe course of acute gastroenteritis. We used the Modified Vesikari Score (MVS). The MVS is a clinical severity score that contains seven equally weighted variables and consists of three categories: mild, 0–8; moderate, 9–10; and severe, ≥11 [19,20]. The seven variables are diarrhoea duration, maximal number of diarrhoea stools per 24-hour period, vomiting duration, maximal number of vomiting episodes per 24-hour period, maximal recorded fever, healthcare provider visits and treatment (Supplement Table 1). We have assessed these variables with baseline data and 7-days follow-up data. We also evaluated referral as a secondary outcome. Referral details concerned referral to secondary paediatric care at baseline or during follow-up. This was treated as a dichotomous variable.

### Sample size calculation

We estimated that 50% of children with, and 20% without, rotavirus infection would have a severe course of acute gastroenteritis [21]. Therefore, we considered that a difference of 30% would be clinically relevant in primary care OOH services. To achieve a power of 80% (two-tailed alpha, 0.05), we required a sample size of 39 patients in each arm, which made a total sample size of 78 patients with acute gastroenteritis to detect this difference. Given an expected loss to follow-up of 5%, we aimed to include 82 patients.

### Data analysis

We described baseline characteristics for the total cohort, for children without a stool sample (i.e. those excluded from analysis), for children with a stool sample (i.e. those included in the analysis) and for children included in the parallel randomised controlled trial to provide insight in possible selection bias. The prevalence (with their 95% CI) of pathogens, both alone and in combination, was calculated for the group of included children who provided stool samples, and also by age groups of 6 to 12 months and ≥1–6 years. This division in subgroups was based on the fact that in children aged below one year the risk on dehydration is the highest as a result of age-related features [22]. Furthermore, the median Ct value, the Ct value range, and the Ct value category were given for each pathogen to detect differences between groups.

The chi-square test was used to evaluate if a statistically significant difference existed in the proportion of children with a severe course of acute gastroenteritis according to the MVS by the presence or absence of rotavirus infection. Subgroup analyses were performed to explore whether a rotavirus Ct value <20, the MVS at baseline, a lower threshold of the MVS (mild versus moderate/severe), or division in age groups influenced the association. Odds ratios (OR) and their 95% confidence intervals (CI) were reported for the primary outcome and the subgroup analyses. Next, we evaluated if differences existed between groups by the presence or absence of other viruses (e.g. adenovirus, astrovirus, enterovirus, norovirus, and sapovirus) if the pathogen had a minimum prevalence of 20%. Meaningful analysis was not possible in smaller samples. We analysed categorical outcomes by chi-square tests. All data were analysed using IBM SPSS Version 25.0 (IBM Corp., Armonk, NY, USA).

## Results

### Patient characteristics

**Figure 1.**
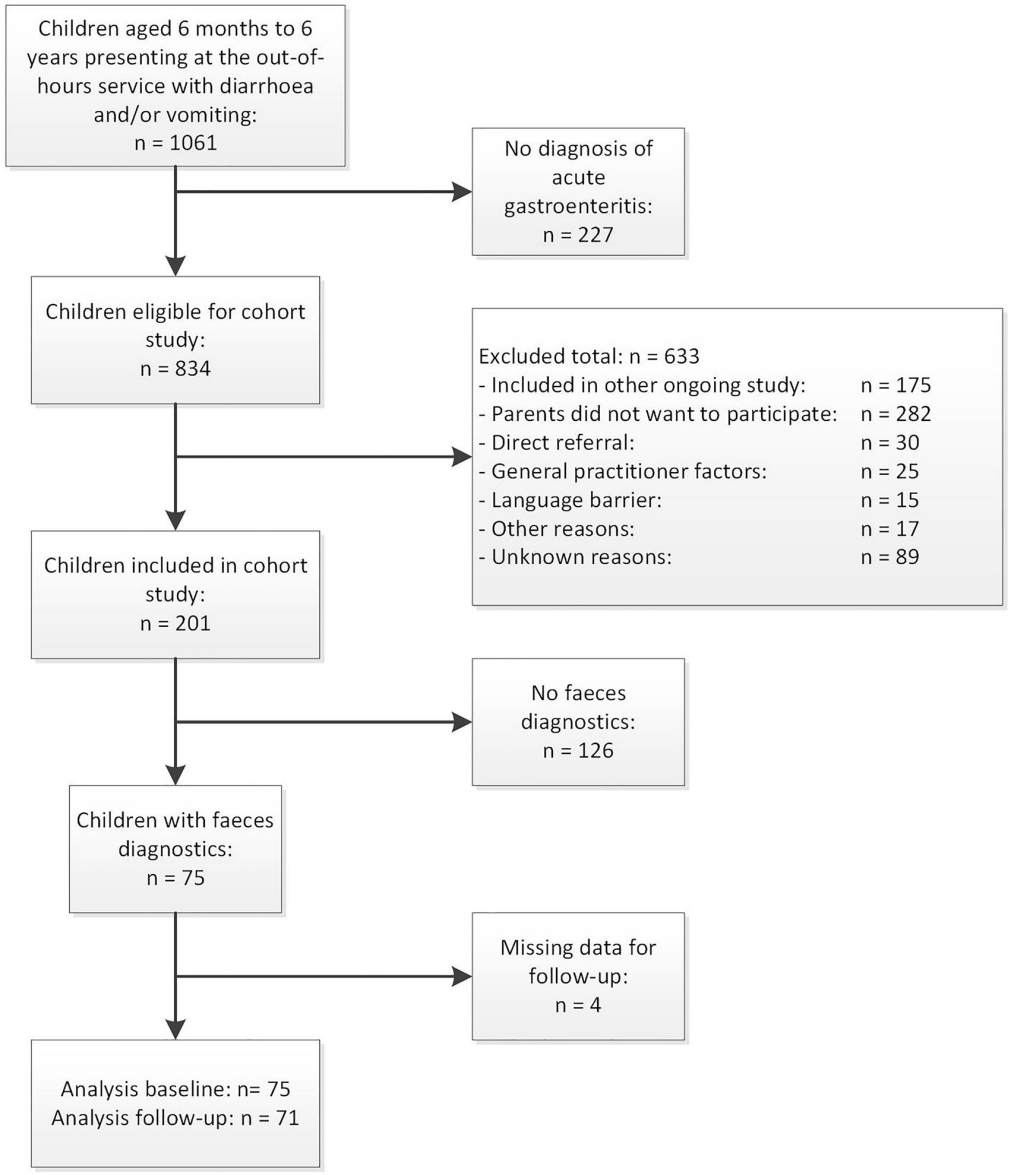
Flowchart of patient selection.

Of the 201 children included in the cohort study, we ultimately enrolled 75 with sufficient stool samples for viral testing. Lack of parental consent or current participation in an ongoing study were the main reasons for exclusion (Figure 1). The median age of the 75 included children was 1.5 years (IQR 0.9–2.0), and 34 (45.3%) were boys (Table 1). At baseline, the 75 patients with stool samples had shorter vomiting durations and lower median frequency of vomiting than the 126 children without stool samples. Children included in the trial vomited more often and had shorter diarrhoea duration (Table 1). Returned diaries contained missing values for 4 children (5.3%), who we, therefore, treated as losses to follow-up, leaving 71 children for the evaluation of illness severity.

**Table 1.** Baseline characteristics of the total cohort, included children, excluded children and children included in the trial.

Characteristics	Total cohort	Included	Excluded	Trial
Number, *N*	201	75	126	191
Gender (male), *N* (%)	99 (49.3)	34 (45.3)	65 (51.6)	92 (48.3)
Age in years, median (IQR)	1.5 (0.9–2.1)	1.5 (0.9–2.0)	1.6 (0.9–2.3)	1.5 (0.9–2.2)
Age categories				
* 6–12 months, N (%)*	52 (25.9)	19 (25.3)	33 (26.2)	52 (27.2)
* ≥1–6 years, N (%)*	149 (74.1)	56 (74.7)	93 (73.8)	139 (72.8)
Vomiting at presentation				
* Duration in hours, median (IQR)^a^*	48.0 (24.0–72.0)	24.0 (1.8–72.0)	48.0 (24.0–72.0)	48.0 (24.0–72.0)
* Frequency in previous 24 h, median (IQR)^b^*	4.0 (2.0–8.0)	2.0 (1.0–4.0)	4.0 (2.0–8.0)	5.0 (4.0–10.0)
Diarrhoea at presentation				
* Duration in hours, median (IQR)^a^*	72.0 (48.0–96.0)	48.0 (17.0–96.0)	48.0 (0.0–96.0)	24.0 (0.0–72.0)
* Frequency in previous 24 h, median (IQR)^b^*	4.0 (2.0–6.0)	3.0 (2.0–6.0)	3.0 (0.0–4.0)	2.0 (0.0–0.5)
Fever at presentation, *N* (%)^c^	51 (25.4)	27 (36.0)	24 (19.0)	49 (25.7)
Referral to secondary care at presentation, n (%)^d^	11 (5.5)	4 (5.3)	7 (5.6)	9 (4.7)

^a^Duration of vomiting and diarrhoea is the duration of symptoms from start to presentation at the primary care OOH and included all children.

^b^Numbers of vomiting and diarrhoeal episodes in the previous 24 h included all children.

^c^Fever was defined as a documented temperature of ≥ 38.0 °C.

^d^Referral to secondary care at presentation was defined as direct referral from the primary care OOH service to secondary care.

IQR = interquartile range.

### Prevalence of pathogens

Among the 75 included children, rotavirus tested positive in 49 (65.3%), whereas other viruses tested positive in numbers ranging from 8 (10.7%) for astrovirus to 32 (42.7%) for adenovirus (Table 2). No bacteria or parasites were found in the included stool samples, and the prevalence of pathogens did not differ by age (Table 2). We also found no virus in four cases (5.3%). Rotavirus was the most frequently detected pathogen and also had the lowest Ct values (median = 16.0), with 75.5% of rotavirus-positive children having a Ct value of <20. More than one virus was present in 43 children (57.3%), with rotavirus and either adenovirus (28.0%) or sapovirus (24.0%) being most frequent. In these pairs, rotavirus still had lower Ct values (Supplement Table 2). We found no coinfection with other viruses in 16 (32.7%) cases of rotavirus infection (Supplement Table 3).

**Table 2.** Prevalence different pathogens and corresponding cycle threshold values (*N* = 75).

Subgroups	*N*	Rotavirus	Adenovirus	Astrovirus	Enterovirus	Norovirus	Sapovirus
All children, *N* (%) (95% CI)	75	49 (65.3) (53.5–76.0)	32 (42.7) (31.3–54.6)	8 (10.7) (4.7–19.9)	23 (30.7) (20.5–42.4)	17 (22.7) (13.8–33.8)	25 (33.3) (22.9–45.2)
Children aged 6–12 months, *N* (%) (95% CI)	19	10 (52.6) (28.9–75.6)	9 (47.4) (24.4–71.1)	2 (10.5) (1.3–33.1)	7 (36.8) (16.3–61.6)	6 (31.6) (12.6–56.6)	4 (21.1) (6.1–45.6)
Children age*d* ≥ 1–6 years, *N* (%) (95% CI)	56	39 (69.6) (55.9–81.2)	23 (41.1) (28.1––55.0)	6 (10.7) (4.0–21.9)	16 (28.6) (17.3–42.2)	11 (19.6) (10.2–32.4)	21 (37.5) (24.9–51.5)
Ct value median		16.0	30.5	21.5	29.0	21.0	25.0
Ct value range		10–37	4–38	8–33	13–38	10–37	10–35
Ct value categories							
<20, *N* (%)^a^		37 (75.5)	5 (15.6)	4 (50.0)	7 (30.4)	8 (47.1)	4 (16.0)
20–30, *N* (%)^a^		11 (22.4)	11 (34.4)	3 (37.5)	8 (34.8)	3 (17.6)	17 (68.0)
>30, *N* (%)^a^		1 (2.0)	16 (50.0)	1 (12.5)	8 (34.8)	6 (35.3)	4 (16.0)

The cycle threshold (Ct) was used to quantify the viral load, with lower values indicating a higher load. There were no significant differences between age groups.

^a^Percentages are calculated with total amount of children with each specific virus infection as denominator.

### Differences between groups

A severe course of acute gastroenteritis was identified in 31 out of 71 children (43.7%). A severe course presented not statistically significantly more often among children with rotavirus (20/47; 42.6%) than among those without rotavirus (11/24; 45.8%) (Table 3). The OR for this was 0.88 (95% CI 0.33–2.36; *p*-value 0.81). Subgroup analyses showed no significant association (Supplement Table 4). Referral rates were comparable between groups (i.e. 15.2% for rotavirus and 14.3% for non-rotavirus) (Table 3). Referral rate and severity did not depend on the presence of one of the viral pathogens detected (Supplement Table 5).

**Table 3.** Differences between pathogens and factors related to the course of acute gastroenteritis.

Outcome	*N*	Total	Rotavirus	No rotavirus	*p*-value
Severe course of acute gastroenteritis, *n*/*N*(%)	71	31/71 (43.7)	20/47 (42.6)	11/24 (45.8)	0.81
Referral at baseline or during follow-up, *n*/*N*(%)	54	8/54 (14.8)	5/33 (15.2)	3/21 (14.3)	0.93

STROBE Statement—Checklist of items that should be included in reports of ***cohort studies***.

## Discussion

### Main findings

We studied 71 children aged 6 months to 6 years presenting to three primary care OOH services with acute gastroenteritis between 2016 and 2018 in the Netherlands. The results provide insight into the proportion of children who have severe courses (43.7%) of acute gastroenteritis among those with and without a rotavirus-positive stool sample. Rotavirus was not only detected most often (65.3%) but also had the lowest Ct values, with about three-quarters of tests being strongly positive. However, we found no statistically significant association between the presence of rotavirus (also with a strongly positive Ct value) and a severe disease course.

### Strengths and limitations

To the best of our knowledge, no study has evaluated the course of acute gastroenteritis due to rotavirus among children presenting to primary care OOH services. The present study benefitted from being conducted prospectively, collecting specific and complete data, and minimising recall error. Another strength is that we evaluated multiple pathogens in stool samples and measured the corresponding Ct values. Identifying many viruses, both alone and in combination, provides further insight into the prevalence and Ct values of the main pathogens in children with acute gastroenteritis in primary OOH care.

A limitation of our study is that we included fewer patients in our primary analysis than required by our sample size calculation (71 rather than 78 children). This was mainly due to lack of parental consent and participation in a parallel randomised controlled trial. At the start of the cohort study, faecal samples were not collected due to logistic reasons. Although we started sampling at a random time point, the patients who collected faeces vomited less often and for a shorter period. Our estimate of a clinically relevant difference of 30% was based on limited evidence from scarce literature in the primary care OOH setting and might be overestimated. However, the differences we found were of no clinical relevance. Indicating that a larger sample might not have overcome the lack of difference found. Another limitation is that viral loads, and thus Ct values, depend on the timing of stool sampling, thereby making the symptom duration a confounder of the measured Ct values, with the possibility of underestimating the association with severe course. Unfortunately, date of sampling was not noted in our study. Finally, we may have preferentially included children with a less severe course of acute gastroenteritis. Children with heavy vomiting and risk for dehydration were included in a parallel randomised controlled trial. When children were very ill and referred to secondary care immediately, parents often did not want to participate in the study beyond that point. Due to this possible selection bias, the associations found between viral pathogen and severe course might be underestimated in case of a true association. These limitations indicate that the data in this study should be interpreted with caution.

### Comparison with existing literature

In line with other studies of children with acute gastroenteritis [2,4,23], over half of the children (65.3%) in our study were infected with rotavirus. More than half (57.3%) also had a coinfection, a common phenomenon [24]. The combination of adenovirus and rotavirus (28.0%) occurred most frequently, consistent with research in which 27.4% of children were shown to be infected with this combination [24]. Sapovirus and adenovirus infections were also frequently present in coinfections but the pathogenicity of these viruses was questionable because their viral loads were often lower than those of the coinfections. We also cannot exclude the possibility that adenovirus was shed into the faeces after a recent respiratory tract infection, instead of being a primary gastroenteritis-causing pathogen. A study among hospitalised children also found adenovirus infection to be relatively common (23%), with most cases present as part of a coinfection (73%). Sapovirus has been reported to be present as a coinfection in only one case [4].

Although there has been some debate about the pathogenicity of enterovirus as a cause of acute gastroenteritis, two recent studies have demonstrated an association [25,26]. Unfortunately, we cannot comment on the pathogenicity of enterovirus or other viruses because we did not perform faecal diagnostic testing on healthy controls. Nevertheless, we found no significant difference between the presence of enterovirus and a severe course of acute gastroenteritis, with most enterovirus-positive cases having high Ct values and being present in coinfections.

We also detected no cases of bacterial or parasitic infection in this study. This is again consistent with existing literature showing that such infections mainly occur in older children and adults [27]. Besides this, an explanation could be that children infected with bacteria or parasites usually present with long-term symptoms and will probably present during regular general practice hours instead of OOH [28]. Therefore, we cannot advocate routine testing for bacterial and parasitic infections in young children with acute severe diarrhoea to a primary care OOH service.

The finding that rotavirus infection was not associated with a severe course of acute gastroenteritis and a higher referral rate is inconsistent with research conducted in hospital settings [3,27]. Several reasons might explain this result. First, there might be no association in low-risk children with acute gastroenteritis. We excluded high-risk children, which might have a higher chance of rotavirus infection and a severe course. Selectively excluding these children might have biased our findings in case of a true relation between rotavirus and severe course. Second, the virus load might influence the association. However, our subgroup analysis showed no association when we included only children with a low Ct value in the rotavirus group. Finally, several clinical scores assess the severity that might have influenced our results [20]. Although the MVS is reliable and valid to measure the severity of acute gastroenteritis in children [20], our finding of a prevalence of severe course of 43.7% is not in line with a prevalence of 10–30% found in previous studies using the MVS [19,20]. In our subgroup analysis where the MVS at baseline was used, the prevalence of a severe course was 19.7%, which is better in line with previous studies.

### Clinical relevance

The presence of a rotavirus-positive stool sample was not associated with an increased OR for a child having a severe course of acute gastroenteritis. This could have implications for the introduction of a point-of-care test or vaccine for rotavirus. It is questionable whether point-of-care testing will offer additional value in the management of these children. In our patient group, the GPs doubt whether the child could have a severe course of acute gastroenteritis and should be referred to the hospital. Point-of-care testing might help the GP in this situation. However, based on our study results, knowing which pathogen causes the disease should not affect management of the GP.

Recently, the *Lancet Global Health* published that there has been a worldwide reduction of 40% in the proportion of children admitted to hospital due to rotavirus infection since the implementation of rotavirus vaccinations [29]. As such, they now advise other countries to introduce this vaccination in their immunisation programmes. In The Netherlands, rotavirus vaccination was planned for children with low birth weights or congenital disorders. New study results of the RIVAR-study showed that the effectivity in the risk groups with low birth weights or congenital disorders is much lower than expected. Therefore, it was decided to postpone vaccination of these risk groups in The Netherlands [30].

## Conclusion

Rotavirus is the most prevalent virus in children with acute gastroenteritis presenting at the OOH service and does not seem to confer an increased risk of a severe course. This is in line with the finding that children with rotavirus do not get referred more often.

## Supplementary Material

Supplemental Tables

## Figures and Tables

**Table:** 

	Item No	Recommendation	Page No
Title and abstract	1	(*a*) Indicate the study’s design with a commonly used term in the title or the abstract	1 (Title page document)
		(*b*) Provide in the abstract an informative and balanced summary of what was done and what was found	1 (Manuscript document)
Introduction			
Background/rationale	2	Explain the scientific background and rationale for the investigation being reported	2/3
Objectives	3	State-specific objectives, including any prespecified hypotheses	3
Methods			
Study design	4	Present key elements of study design early in the paper	3
Setting	5	Describe the setting, locations, and relevant dates, including periods of recruitment, exposure, follow-up, and data collection	4
Participants	6	(*a*) Give the eligibility criteria and the sources and methods of selection of participants. Describe methods of follow-up	4
		(*b*) For matched studies, give matching criteria and number of exposed and unexposed	n/a
Variables	7	Clearly define all outcomes, exposures, predictors, potential confounders, and effect modifiers. Give diagnostic criteria, if applicable	5/6
Data sources/ measurement	8*	For each variable of interest, give sources of data and details of assessment methods (measurement). Describe comparability of assessment methods if there is more than one group	4/5
Bias	9	Describe any efforts to address potential sources of bias	4
Study size	10	Explain how the study size was arrived at	6
Quantitative variables	11	Explain how quantitative variables were handled in the analyses. If applicable, describe which groupings were chosen and why	6/7
Statistical methods	12	(*a*) Describe all statistical methods, including those used to control for confounding	6/7
		(*b*) Describe any methods used to examine subgroups and interactions	6/7
		(*c*) Explain how missing data were addressed	6/7
		(*d*) If applicable, explain how loss to follow-up was addressed	6/7
		(*e*) Describe any sensitivity analyses	n/a
Results			
Participants	13*	(a) Report numbers of individuals at each stage of study—e.g. numbers potentially eligible, examined for eligibility, confirmed eligible, included in the study, completing follow-up, and analysed	7
		(b) Give reasons for non-participation at each stage	7
		(c) Consider use of a flow diagram	See Flowchart
Descriptive data	14*	(a) Give characteristics of study participants (e.g. demographic, clinical, social) and information on exposures and potential confounders	7
		(b) Indicate number of participants with missing data for each variable of interest	7
		(c) Summarise follow-up time (e.g. average and total amount)	n/a
Outcome data	15*	Report numbers of outcome events or summary measures over time	8

** **Main results	16	(*a*) Give unadjusted estimates and, if applicable, confounder-adjusted estimates and their precision (e.g. 95% confidence interval). Make clear which confounders were adjusted for and why they were included	8
		(*b*) Report category boundaries when continuous variables were categorised	8
		(*c*) If relevant, consider translating estimates of relative risk into absolute risk for a meaningful time period	n/a
Other analyses	17	Report other analyses done—e.g. analyses of subgroups and interactions, and sensitivity analyses	8
Discussion			
Key results	18	Summarise key results with reference to study objectives	9
Limitations	19	Discuss limitations of the study, taking into account sources of potential bias or imprecision. Discuss both direction and magnitude of any potential bias	9/10
Interpretation	20	Give a cautious overall interpretation of results considering objectives, limitations, multiplicity of analyses, results from similar studies, and other relevant evidence	9–12
Generalisability	21	Discuss the generalisability (external validity) of the study results	12
Other information			
Funding	22	Give the source of funding and the role of the funders for the present study and, if applicable, for the original study on which the present article is based	1 (Title page document)

*Give information separately for exposed and unexposed groups.

**Note:** An Explanation and Elaboration article discusses each checklist item and gives methodological background and published examples of transparent reporting. The STROBE checklist is best used in conjunction with this article (freely available on the Web sites of PLoS Medicine at http://www.plosmedicine.org/, Annals of Internal Medicine at http://www.annals.org/, and Epidemiology at http://www.epidem.com/). Information on the STROBE Initiative is available at http://www.strobe-statement.org.
