# Supplementary material for: Rotavirus and illness severity in children presenting with acute gastroenteritis at the primary care out-of-hours service
Source: Eur J Gen Pract. 2021 Dec 13;27(1):346–53. doi: 10.1080/13814788.2021.2011205 (PMC8741236; doi:10.1080/13814788.2021.2011205)
Supplement: Supplemental Tables [file IGEN_A_2011205_SM8247.docx]

| **Supplement Table 1.** Modified Vesikari Score (MVS) Components | | | | |
| --- | --- | --- | --- | --- |
|  | **0 Points** | **1 Point** | **2 Points** | **3 Points** |
| Diarrhoea duration, days | 0 | 1-4 | 5 | ≥ 6 |
| Maximal no. of diarrhoeal stools per 24-h period | 0 | 1-3 | 4-5 | ≥ 6 |
| Vomiting duration, days | 0 | 1 | 2 | ≥ 3 |
| Maximal no. of vomiting episodes per 24-h period | 0 | 1 | 2-4 | ≥ 5 |
| Maximal recorded fever, rectal | < 37.0 | 37.1-38.4 | 38.5-38.9 | ≥ 39.0 |
| Health care provider visits | None |  | Outpatient^a^ | ED^b^ |
| Treatment | None | Rehydration | Hospitalization |  |
| ^a^ Community-based health care provider visit related to vomiting, diarrhoea, fever, or fluid refusal  ^b^ ED health care provider visit related to vomiting, diarrhoea, fever, or fluid refusal  Categories: mild, 0 – 8; moderate, 9 – 10; severe, ≥ 11 | | | | |

| **Supplement Table 2.** Combinations of pathogens: prevalence and Cycle threshold ranges (*N* = 75) | | | | | | |
| --- | --- | --- | --- | --- | --- | --- |
| **Virus 1** | **Virus 2** | ***N* (%)** | **Virus 1** | | **Virus 2** | |
|  |  |  | **median** | **Ct range** | **median** | **Ct range** |
| Adenovirus | Rotavirus | 21 (28.0) | 33.0 | 25–38 | 14.0 | 10–37 |
| Rotavirus | Sapovirus | 18 (24.0) | 14.5 | 10–29 | 25.0 | 19–35 |
| Rotavirus | Enterovirus | 16 (21.3) | 13.0 | 10–37 | 30.0 | 13–38 |
| Adenovirus | Sapovirus | 15 (20.0) | 30.0 | 23–38 | 25.0 | 10–33 |
| Adenovirus | Enterovirus | 15 (20.0) | 32.0 | 25–38 | 30.0 | 13–36 |
| Adenovirus | Norovirus | 10 (13.3) | 26.5 | 6–36 | 30.5 | 16–37 |
| Enterovirus | Norovirus | 7 (9.3) | 30.0 | 16–33 | 32.0 | 14–37 |
| Enterovirus | Sapovirus | 7 (9.3) | 23.0 | 13–34 | 28.0 | 24–33 |
| Norovirus | Rotavirus | 7 (9.3) | 35.0 | 21–37 | 15.0 | 10–29 |
| Adenovirus | Astrovirus | 5 (6.7) | 27.0 | 26–37 | 18.0 | 8–33 |
| Astrovirus | Rotavirus | 5 (6.7) | 27.0 | 8–33 | 15.0 | 12–18 |
| Norovirus | Sapovirus | 5 (6.7) | 21.0 | 10–36 | 25.0 | 24–29 |
| Astrovirus | Enterovirus | 4 (5.3) | 13.0 | 8–28 | 27.0 | 23–33 |
| Astrovirus | Norovirus | 3 (4.0) | 18.0 | 8–25 | 30.0 | 16–32 |
| Astrovirus | Sapovirus | 3 (4.0) | 25.0 | 8–28 | 25.0 | 24–25 |
| The cycle threshold (Ct) was used to quantify the viral load, with lower values indicating a higher load. | | | | | | |

| **Supplement Table 3.** Prevalence and Cycle threshold ranges for single infections (***N*** = 75) | | | |
| --- | --- | --- | --- |
| **Pathogen(s)** | ***N* (%)^a^** | **Median** | **Ct range** |
| Rotavirus | 16 (32.7) | 18.5 | 10–27 |
| Norovirus | 3 (17.6) | 13.0 | 10–16 |
| Adenovirus | 3 (9.4) | 15.0 | 4–17 |
| Astrovirus | 1 (12.5) | 13.0 | 13 |
| Enterovirus | 3 (13.0) | 18.0 | 13–19 |
| Sapovirus | 2 (8.0) | 24.0 | 19–29 |
| The cycle threshold (Ct) was used to quantify the viral load, with lower values indicating a higher load.  ^a^ Denominator is total of infections of each specific virus. | | | |

| **Supplement Table 4.** Subgroup analyses of the association between rotavirus and a severe course of acute gastroenteritis | | | |
| --- | --- | --- | --- |
| **Subgroup analysis** | **Rotavirus, *N* (%)** | **No rotavirus, n (%)** | **OR (95% CI)** |
| Ct value <20^a^ | 16/35 (45.7) | 15/36 (41.7) | 1.18 (0.46 – 3.01) |
| Severe MVS at baseline^b^ | 7/47 (14.9) | 1/24 (4.2) | 4.03 (0.47 – 34.80) |
| Severe course defined with a lower threshold of the MVS^c^ | 32/48 (66.7) | 15/23 (65.2) | 1.07 (0.37 – 3.04) |
| Severe course in age 6 to 12 months | 2/9 (22.2) | 4/8 (50.0) | 0.29 (0.04 – 2.32) |
| Severe course in age ≥ 1 to 6 years | 18/38 (47.4) | 7/16 (43.8) | 1.16 (0.36 – 3.75) |
| **P*-value < 0.05  ^a^ Association between a rotavirus infection with a Ct value < 20 and severe course  ^b^ Severe MVS defined with the information available at baseline and the association with rotavirus infection  ^c^ Division in MVS categories mild vs moderate/severe and the association with rotavirus infection | | | |

| **Supplemental Table 5.** Pathogens and their association with a severe course and referral of acute gastroenteritis | | | |
| --- | --- | --- | --- |
| **Pathogen** | **Severe course of acute gastroenteritis, *n/N* (%)** | **No severe course of acute gastroenteritis, *n/N* (%)** | **Referral at baseline or during follow-up, *n/N* (%)** |
| Rotavirus | 20/47 (42.6) | 27/47 (57.4) | 5/33 (15.2) |
| Adenovirus | 17/30 (56.7) | 13/30 (43.3) | 4/22 (18.2) |
| Enterovirus | 14/23 (60.9) | 9/23 (39.1) | 3/15 (20.0) |
| Norovirus | 5/16 (31.3) | 11/16 (68.7) | 2/11 (18.2) |
| Sapovirus | 10/24 (41.7) | 14/24 (58.3) | 2/20 (10.0) |
| **P*-value < 0.05 | | | |
